# Toward comprehensive palliative care through the vital role of culturally adapted spiritual interventions

**DOI:** 10.1017/S1478951524001780

**Published:** 2025-01-21

**Authors:** Riza Amalia, Ahmad Razak, Lutfi Hidayati Fauziah, Ria Angin, Wikan Galuh Widyarto, Tamama Rofiqoh, Hanik Mufaridah

**Affiliations:** 1 Department of Guidance & Counseling, University of Muhammadiyah Sampit, Kotawaringin Timur, Central Kalimantan, Indonesia; 2 Guidance & Counseling Department, Universitas Negeri Malang, Malang, East Java, Indonesia; 3 Faculty of Psychology, Universitas Negeri Makassar, Makassar, South Sulawesi, Indonesia; 4 Faculty of Psychology, Universitas Merdeka Malang, Malang, East Java, Indonesia; 5 Government Studies, University of Muhammadiyah Jember, Jember, East Java, Indonesia; 6 Department of Islamic Guidance & Counseling, Universitas Islam Negeri Sayyid Ali Rahmatullah Tulungagung, Tulungagung, East Java, Indonesia; 7 Department of Guidance & Counseling, Riau Kepulauan University, Batam, Kepulauan Riau, Indonesia; 8 Faculty of Da’wa, Ibrahimy University, Situbondo, East Java, Indonesia

Dear Editor,

We read with great interest on recent published scoping review on spiritual care interventions in palliative care titled, “Spiritual care interventions for palliative care patients: A scoping review” by Jaman-Mewes et al, this article underscores a critical but often overlooked aspect of patient care: the spiritual dimension (Jaman-Mewes et al. 2024). As the global healthcare community continues to seek comprehensive approaches to alleviate suffering in palliative care, it is essential to address two pivotal factors that can significantly enhance the effectiveness of spiritual care interventions: cultural sensitivity and the integration of spiritual care providers into multidisciplinary teams.

One of the most pressing needs identified in the review is the adaptation of spiritual care interventions to suit diverse populations. Spiritual needs are deeply personal and are shaped by a wide array of cultural, religious, and spiritual beliefs (Russo-Netzer 2017). Therefore, the effectiveness of spiritual care is contingent upon its ability to be culturally sensitive and adaptive. In palliative care settings, patients often face existential distress and seek comfort through familiar spiritual and cultural practices. However, a one-size-fits-all approach to spiritual care fails to acknowledge the rich diversity of patients’ backgrounds and beliefs. For instance, spiritual care that resonates with a patient in a Western context may not be suitable for someone from an Asian or Middle Eastern background, where different religious traditions and spiritual practices prevail. In the case of Muslim patients (Ghamian 2024), for example, spiritual care may include practices such as reading or listening to verses from the Quran, engaging in prayer (Salah), or ensuring that the patient has access to a clean environment to perform ablution (Wudu). Additionally, the presence of an Imam or a Muslim chaplain can provide comfort and guidance, aligning the spiritual care provided with Islamic beliefs and practices. This cultural and religious sensitivity ensures that spiritual care is both meaningful and respectful, providing solace to patients in alignment with their faith.

To improve the acceptance and effectiveness of spiritual care interventions globally, it is imperative that healthcare providers are trained to recognize and respect the unique spiritual needs of each patient. Developing culturally adapted spiritual care models, incorporating local religious and spiritual leaders (Oxhandler et al. 2024), and tailoring interventions to align with patients’ beliefs can greatly enhance the therapeutic impact of these interventions. Culturally sensitive spiritual care not only fosters a deeper connection between patients and providers but also validates the patients’ beliefs, offering a sense of peace and comfort during their most vulnerable moments. While cultural adaptation is vital, the integration of spiritual care practitioners into multidisciplinary palliative care teams is equally crucial. Spiritual care should not exist in isolation; rather, it should be an integral part of a holistic care model that includes medical, psychological, and social support. The collaborative approach involving doctors, nurses, psychologists, social workers, and spiritual care providers can address the multifaceted needs of patients, ensuring that care is comprehensive and personalized.

Spiritual care providers bring a unique set of skills that complement the medical expertise of other team members (de Diego-cordero et al. 2023). They offer a compassionate presence, facilitate meaningful conversations, and provide emotional and spiritual support that medical professionals may not be equipped to deliver. By working together, healthcare professionals and spiritual care providers can create a more cohesive care plan that honors the patient’s values, beliefs, and wishes.

Furthermore, integrating spiritual care into multidisciplinary teams can enhance communication among providers, reduce fragmentation of care, and ensure that the patient’s spiritual needs are consistently addressed throughout their palliative journey. This collaborative model not only improves patient satisfaction but also enriches the overall quality of care, as each team member contributes to a shared goal of holistic healing.
